# Genomic analysis of inter-hospital transmission of vancomycin-resistant *Enterococcus faecium* sequence type 80 isolated during an outbreak in Hiroshima, Japan

**DOI:** 10.1128/aac.01716-23

**Published:** 2024-03-20

**Authors:** Takaya Segawa, Kanako Masuda, Junzo Hisatsune, Kasumi Ishida-Kuroki, Yo Sugawara, Masao Kuwabara, Hideki Nishikawa, Takahiro Hiratsuka, Tatsuaki Aota, Yasuo Tao, Yoshimi Iwahashi, Kuniko Ueda, Kaori Mae, Ken Masumoto, Hiroki Kitagawa, Hitoshi Komatsuzawa, Hiroki Ohge, Motoyuki Sugai

**Affiliations:** 1 Antimicrobial Resistance Research Center, National Institute of Infectious Diseases, Higashimurayama, Japan; 2 Hiroshima Prefectural Center for Disease Control and Prevention, Hiroshima, Japan; 3 Project Research Center for Nosocomial Infectious Diseases, Hiroshima University, Hiroshima, Japan; 4 Department of Antimicrobial Resistance, Hiroshima University Graduate School of Biomedical & Health Sciences, Hiroshima, Japan; 5 Hiroshima Prefectural Technology Research Institute, Public Health and Environment Center, Hiroshima, Japan; 6 Hiroshima City Institute of Public Health, Hiroshima, Japan; 7 Hiroshima City Public Health Center, Hiroshima, Japan; 8 Hiroshima City Medical Association Clinical Laboratory, Hiroshima, Japan; 9 Department of Infectious Diseases, Hiroshima University Hospital, Hiroshima, Japan; 10 Department of Bacteriology, Hiroshima University Graduate School of Biomedical and Health Sciences, Hiroshima, Japan; University of Fribourg, Fribourg, Switzerland

**Keywords:** vancomycin-resistant enterococci, *Enterococcus faecium *ST80, *vanA*, outbreak, genomic analysis, multilocus sequence typing

## Abstract

Outbreaks caused by vancomycin-resistant enterococci that transcend jurisdictional boundaries are occurring worldwide. This study focused on a vancomycin-resistant enterococcus outbreak that occurred between 2018 and 2021 across two cities in Hiroshima, Japan. The study involved genetic and phylogenetic analyses using whole-genome sequencing of 103 isolates of vancomycin-resistant enterococci to identify the source and transmission routes of the outbreak. Phylogenetic analysis was performed using core genome multilocus sequence typing and core single-nucleotide polymorphisms; infection routes between hospitals were inferred using BadTrIP. The outbreak was caused by *Enterococcus faecium* sequence type (ST) 80 carrying the *vanA* plasmid, which was derived from strain A10290 isolated in India. Of the 103 isolates, 93 were *E. faecium* ST80 transmitted across hospitals. The circular *vanA* plasmid of the Hiroshima isolates was similar to the *vanA* plasmid of strain A10290 and transferred from *E. faecium* ST80 to other STs of *E. faecium* and other *Enterococcus* species by conjugation. The inferred transmission routes across hospitals suggest the existence of a central hospital serving as a hub, propagating vancomycin-resistant enterococci to multiple hospitals. Our study highlights the importance of early intervention at the key central hospital to prevent the spread of the infection to small medical facilities, such as nursing homes, with limited medical resources and a high number of vulnerable individuals.

## INTRODUCTION

**Fig 1:**
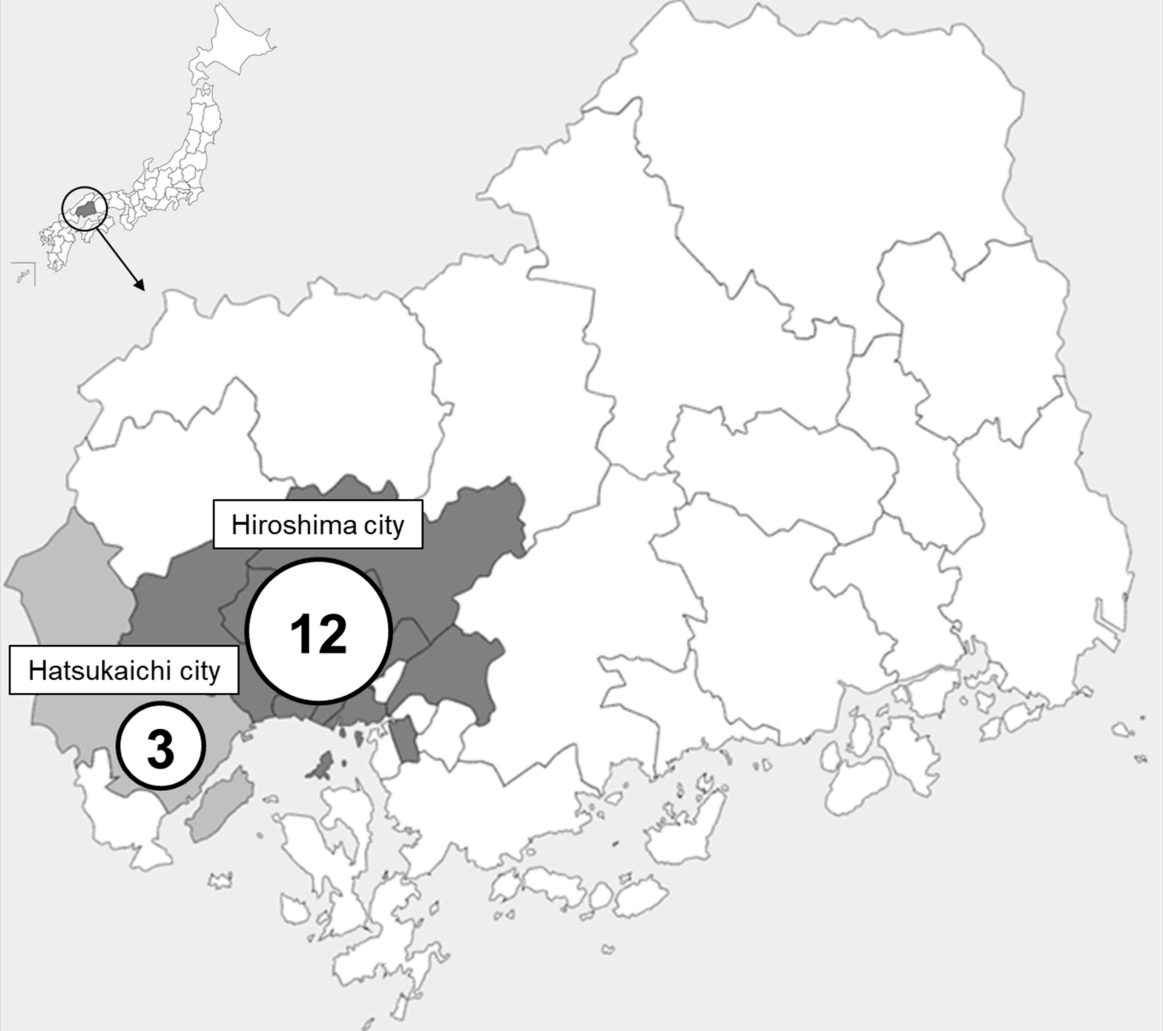
Regions and number of hospitals in which vancomycin-resistant enterococci (VRE) were detected from 2018 to 2021. VRE were isolated from the neighboring cities of Hiroshima and Hatsukaichi in Hiroshima Prefecture, western Japan. Hiroshima city is the prefectural capital (https://n.freemap.jp/). Numbers in the circles indicate the number of hospitals in which VRE were detected in each city.

**Fig 2:**
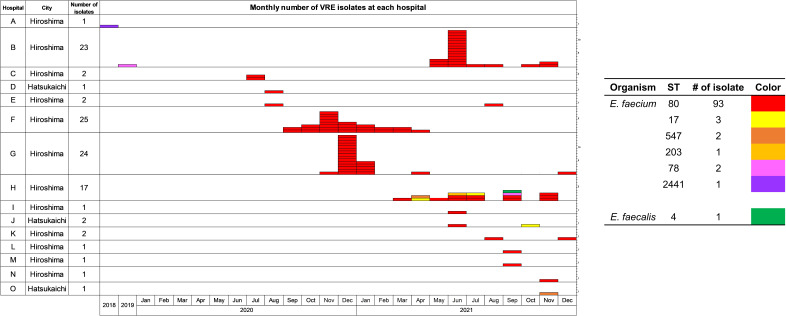
Number of vancomycin-resistant enterococci (VRE) isolated monthly at each hospital. This graph shows the number of VRE isolated per month at each hospital (A–O). Each tile indicates a bacterial strain and is shown in the following colors according to the bacterial species and sequence type; red, *E. faecium* ST80; yellow, *E. faecium* ST17; dark orange, *E. faecium* ST547; orange, *E. faecium* ST203; pink, *E. faecium* ST78; purple, *E. faecium* ST 2441; and green, *E. faecalis* ST4.

Enterococci are commensal bacteria in the gastrointestinal tract of healthy individuals that can be nosocomial pathogens causing urinary tract infections, bacteremia, endocarditis, meningitis, and other infections in compromised hosts. Most enterococci isolated in clinical settings are *Enterococcus faecium* and *Enterococcus faecalis*, with *E. faecium* tending to be more multidrug-resistant than *E. faecalis*. To treat the infections, ampicillin is the first-choice antibiotic if *E. faecium* is susceptible (1). However, vancomycin, an active glycopeptide antibiotic against Gram-positive bacteria, becomes essential as most *E. faecium* bacteria in Japan are ampicillin-resistant (1–6). Outbreaks of nosocomial infections caused by vancomycin-resistant enterococci (VRE) have recently increased in Europe and Australia, as *E. faecium* belongs to clonal complex 17 (CC17) (7–11). *E. faecium* CC17 has adapted to the hospital environment by acquiring antimicrobial resistance genes, pathogenicity islands, and other mobile genetic elements (12, 13). Outbreaks were caused by *E. faecium* CC17 with the vancomycin-resistance genes *vanA* and *vanB* (7–11, 14–19). Both *vanA* and *vanB* encode ligases that contribute to vancomycin resistance by substituting the D–Ala–D–Ala peptide in peptidoglycans with D–Ala–D–Lac, and these carriers have been reported worldwide, although the *vanA* carrier is more prevalent (20). In Japan, VRE infections have shown a recent increasing trend according to the National Epidemiological Surveillance of Infectious Diseases (6, 21, 22). In 2019, a widespread outbreak of vancomycin-resistant *E. faecium* ST1421 (CC17) occurred in Aomori that extended beyond the jurisdiction of health authorities (23). The numerical target for patient numbers with VRE infection was set for the first time in the Japanese National Action Plan on AMR in 2023 (24), emphasizing the need for strengthened measures. In this study, a large outbreak of VRE extending beyond the jurisdiction of health authorities in Hiroshima between 2018 and 2021 (Fig. 1 and 2) was genetically and phylogenetically analyzed using whole-genome sequencing to identify the source and transmission routes of VRE (Fig. S1). The genomic sequences of the isolates were characterized to estimate the factors contributing to the outbreak, as well as the routes of transmission and other factors that could help prevent the spread of VRE.

## RESULTS

### Genetic characterization of *E. faecium* isolated in Hiroshima

Whole-genome sequencing of VRE isolated in Hiroshima was used to determine the organism species and sequence type (ST) and detect the antimicrobial resistance genes present. The 103 isolates were as follows: 102 strains were *E. faecium* (ST80, 93 strains; ST17, three strains; ST78, two strains; ST547, two strains; ST203, one strain; ST 2441, one strain), and one was *E. faecalis* ST4, revealing that the majority of the isolates were *E. faecium* ST80 (Table S1).

All organisms harbored 10–19 antimicrobial resistance genes and a *vanA* gene cluster (Table S1). The common antimicrobial resistance genes in *E. faecium* were *aac(6’)-Ii* (aminoglycoside resistance), *efmA* (macrolide and fluoroquinolone resistance), and *msrC* (macrolide resistance), while most genes also harbored aminoglycoside resistance genes, such as *aac(6’)-Ie-aph(2’’)-Ia*, *aad (6*), and *aph(3’)-IIIa*, and tetracycline resistance genes, such as *tet*(L), *tet*(S), and *tet*(U).

### Comparison of *E. faecium* ST80 between Hiroshima and other countries using core genome multilocus sequence typing (MLST)

**Fig 3:**
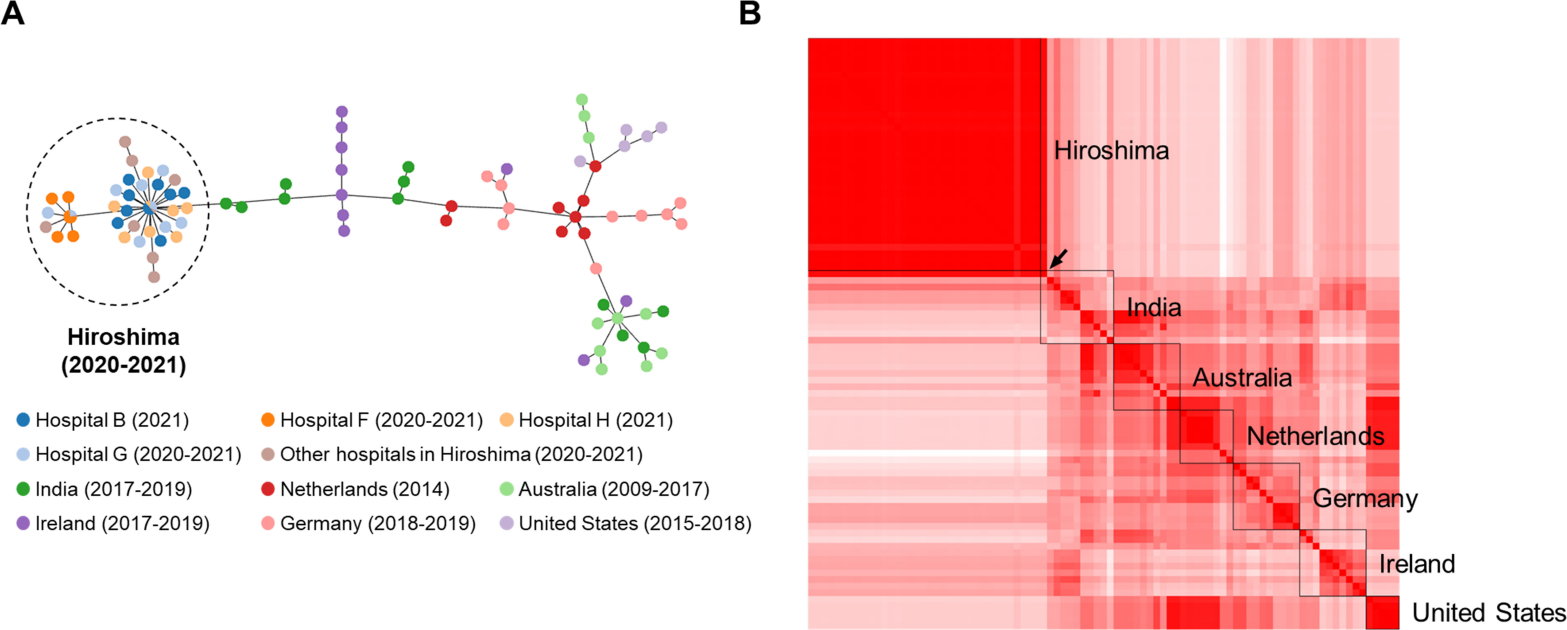
Comparison of *E. faecium* ST80 isolated in Japan and other countries by core genome MLST. The core genome MLST of *E. faecium* ST80 isolates from Hiroshima, India, the Netherlands, Ireland, Germany, Australia, and the United States was analyzed using chewBBACA and visualized on PHYLOViZ Online. A. A minimum-spanning tree was constructed based on the core genome MLST analysis results. Each circle represents an isolate, and the color indicates its location. B. Distance matrix using the same data set as in panel A. The squares with the closest distance are shown in red, and those with the farthest distance are shown in white. Black squares surround each country. The black arrow indicates the isolates from India that are considered closely related to the isolates from Hiroshima.

MLST of the Hiroshima VRE isolates revealed that most VRE isolates were *E. faecium* ST80 (Table S1). As outbreaks caused by ST80 have occurred in Europe and Australia (7–11, 15–19), we determined the origin of Hiroshima isolates using core genome MLST performed with chewBBACA and the sequence reads of *E. faecium* ST80 isolated from Hiroshima (2018–2021), India (2017–2019), the Netherlands (2014), Australia (2009–2017), Ireland (2017–2019), Germany (2018–2019), and the United States (2015–2018) that were deposited in the NCBI (Table S2; Fig. 3). The minimum spanning tree from the core genome MLST showed that all ST80 isolates from the 15 hospitals in Hiroshima clustered in one group and differed from the strains isolated in Europe, Australia, and the United States but were similar to strains found in India (Fig. 3A). A distance matrix was also obtained using these data sets and compared among countries, suggesting that only one strain from India (A10290 strain; GenBank ID, CP059755.1) was similar to the Hiroshima isolates (Fig. 3B).

### Single-nucleotide polymorphism (SNP) analysis between Hiroshima and Indian isolates

As the Indian strain A10290 and the Hiroshima isolates were closely related, we generated a core SNP alignment between eight Indian strains and the 103 Hiroshima isolates using the chromosome sequence of A10290 (CP059755.1) as a reference (Fig. S2). Except for A10290, the other seven Indian strains were in different clades from the Hiroshima isolates, and the number of SNPs between their clades ranged from 110 to 271. Only the Indian A10290 strain was included in the same clade as the Hiroshima strain, and the number of SNPs among the strains in the clade was 0–19, suggesting that Hiroshima isolates were related to A10290.

### Prediction of the transmission route of *E. faecium* ST80 among hospitals in Hiroshima

**Fig 4:**
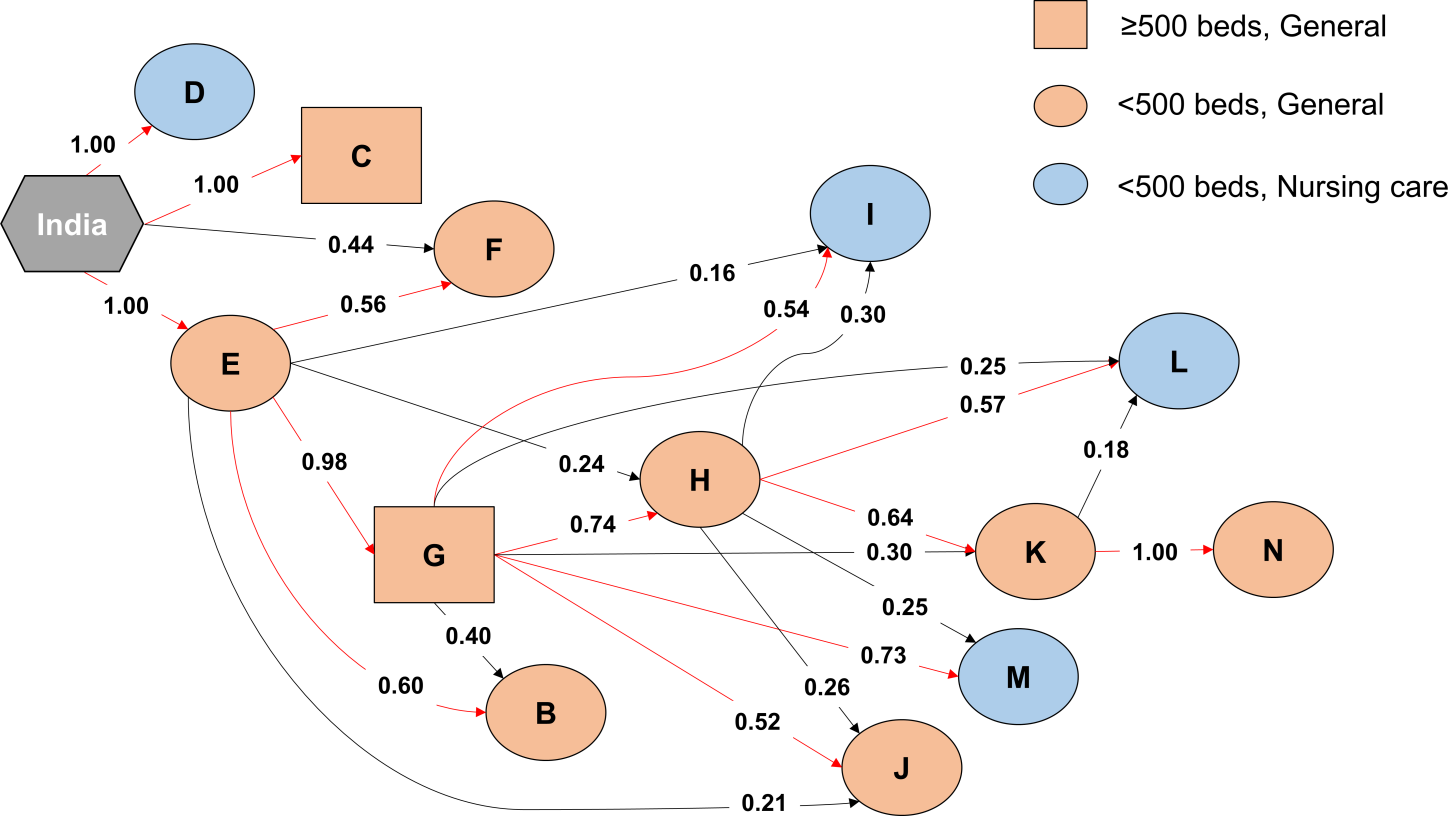
Inference of transmission routes between hospitals in Hiroshima. The transmission of ST80 among hospitals in Hiroshima (B–N) (that began in India) was inferred using BadTrIP. Squares indicate more than 500 beds, and circles indicate less than 500 beds. Orange indicates general care facilities, and blue indicates nursing care facilities. The arrows between hospitals indicate the inferred routes of transmission, with posterior probabilities calculated using the Bayesian approach.

The results of SNP analysis between isolates from Hiroshima and India suggested that the Hiroshima isolates were derived from A10290 (Fig. S2). To infer the events of VRE transmission, we used the Bayesian BadTrIP approach to accurately determine how this strain spread among hospitals in Hiroshima based on the isolation date and SNPs. Hiroshima city and Hatsukaichi city are located next to each other, and the maximum distance between the hospitals in these cities is approximately 20 km. In the reconstructed transmission routes in Hiroshima, there was no spread from Hospital C to the other hospitals where *vanA*-positive ST80 was first isolated during the outbreak (Fig. 4). Similarly, no spread was observed in Hospital D or F; however, it was predicted that ST80 could have spread through Hospital E and then further through large general hospitals, such as Hospital G, before eventually spreading to small- and medium-sized nursing care and general hospitals (Fig. 4).

### Comparison of the plasmid sequences carrying *vanA* in Hiroshima isolates with pA10290 isolated in India

**Fig 5:**
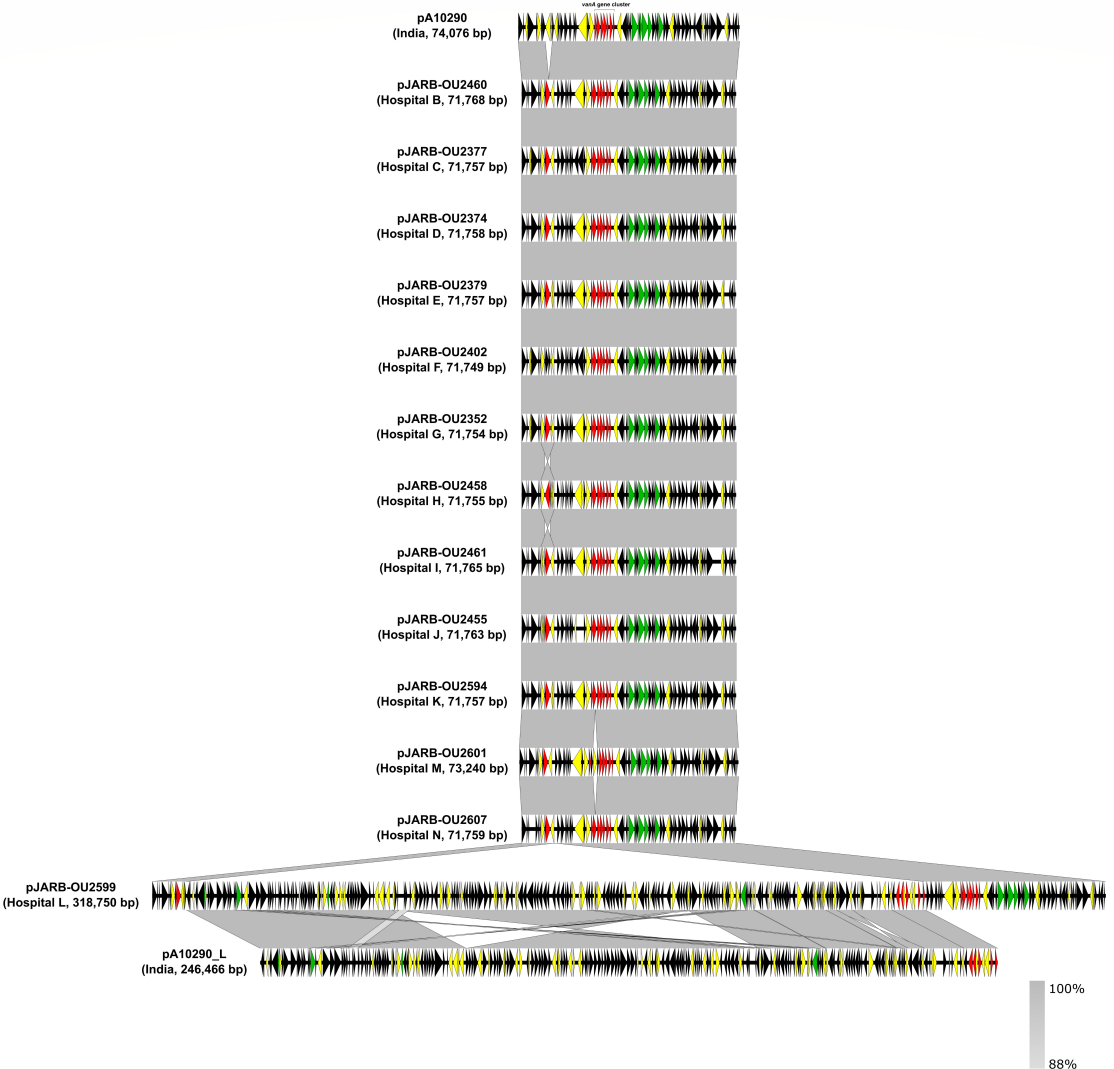
Comparison of the *vanA*-positive plasmid gene structure of A10290 and Hiroshima isolates. The complete genome sequences from thirteen representative *E. faecium* isolates from Hiroshima were determined, and the *vanA*-positive plasmids were compared to the Indian plasmids (pA10290 and pA10290_L) using nucleotide BLAST. The open-reading frames are indicated by arrows, and genes are shown in different colors: red, antimicrobial resistance genes; yellow, IS/transposons; and green, conjugation-related genes. The homology between the strains is shown in dark gray.

The *E. faecium* ST80 isolated in Hiroshima was closely related to strain A10290 from India that carried the *vanA* gene cluster on its linear plasmid, pA10290 (74,0746 bp, accession number CP059757.1). Genome sequencing analysis of the *E. faecium* ST80 isolated in Hiroshima showed that all strains had the *vanA* gene cluster; therefore, the complete genome sequences of the thirteen representative strains isolated from different hospitals were determined by the hybrid assembly of short-read (Illumina) and long-read (Oxford Nanopore) sequences to confirm whether the *vanA* gene was located on the pA10290-like plasmid (Fig. 5). This analysis revealed that the complete nucleotide sequences of the *vanA* gene cluster harbored plasmids (71,755–318,750 bp) in the thirteen strains (Fig. 5). The comparison of the thirteen plasmids with *vanA*-positive pA10290 (CP059757.1) by BLAST showed that, except for pJARB-OU2599, twelve plasmids had an almost identical DNA sequence (Fig. 5). The pJARB-OU2599 plasmid had a sequence corresponding to those of both pA10290 and pA10290_L (another plasmid harbored by A10290, 246,466 bp, accession number CP059756.1; Fig. 5). The pA10290_L-like plasmids were found in clinical isolates of *E. faecium* ST80 in Hiroshima (coverage ≥90%; Table S1). Most *vanA* plasmids contain the aminoglycoside O-phosphotransferase gene *aph(2)-Ia* as an antimicrobial resistance gene. However, the gene was truncated by insertion or mutation in pA10290 and pJARB-OU2402. Other genes encoding transposons and hypothetical proteins were also truncated by mutations in their plasmids (Fig. 5). The remaining 90 Hiroshima isolates harbored *vanA* gene clusters; therefore, the sequence reads of isolates were aligned to the pA10290 complete sequence to determine if these strains carry pA10290-like plasmids. We found that 100 of the 103 isolates carried pA10290-like plasmids (pA10290 positive, coverage ≥90%; Table 1; Table S1) as well as various STs of *E. faecium* and *E. faecalis* (Table 1). The *E. faecalis* plasmid had nearly the same genetic composition as that of pA10290 (Fig. S3). In addition, only one isolate carried the linear plasmid pELF2 (25), which was reported in Japan, and the sequence reads of the two *E. faecium* ST78 isolates were not mapped (Table 1).

**TABLE 1:** Predicted plasmids in enterococci isolated from Hiroshima

Species	ST	Reference for mapping			No mapping	Total
		pA10290	pELF1	pELF2		
*E. faecium*	80	93				93
	17	3				3
	547	2				2
	203	1				1
	78				2	2
	2441			1		1
*E. faecalis*	4	1				1

### pA10290-like plasmid can transfer to other enterococci STs and species

In the Hiroshima isolates, *vanA*-positive plasmids were found in multiple STs of *E. faecium* and *E. faecalis*, suggesting that the plasmids were transferred over various STs of *E. faecium* and other species. The efficiency of conjugational transfer was estimated to confirm whether plasmid conjugation contributed to the outbreak (Table 2). For filter mating, *E. faecium* ST80 carrying the pA10290-like plasmid (JARB-OU2369) was used as the donor, and erythromycin-susceptible strains of *E. faecium* ST17, *E. faecium* ST547, *E. faecalis* ST476, *E. avium*, and *E. gallinarum* were used as the recipients, resulting in frequencies of conjugation of the pA10290-like plasmid of 1 × 10^−5^, 1.1 × 10^−4^, 1.8 × 10^−6^, 0.8 × 10^−7^, and 1.1 × 10^−4^, respectively (Table 2). We also performed mating experiments in the broth, which resulted in a conjugation frequency of 1.4 × 10^−6^ for *E. gallinarum*; however, no conjugation transfer was observed in *E. faecium*, *E. faecalis*, and *E. avium* (Table 2).

**TABLE 2:** Frequency of *vanA*-positive plasmid conjugation in Hiroshima isolates

Recipient strain	Species	ST	Conjugation frequency from JARB-OU2369 (*E. faecium* ST80)	
			On filter	In broth
JARB-HU0742	*E. faecium*	17	1 ± 0.6 × 10^−5^	N.D.
JARB-HU0749	*E. faecium*	547	1.1 ± 1.4 × 10^−4^	N.D.
JARB-HU0782	*E. avium*	−	0.8 ± 1.4 × 10^−7^	N.D.
JARB-HU0796	*E. faecalis*	476	1.8 ± 2.8 × 10^−6^	N.D.
JARB-HU0816	*E. gallinarum*	−	1.1 ± 1 × 10^−4^	1.4 ± 2.4 × 10^−6^

### The pA10290-like plasmid is circular, not linear

**Fig 6:**
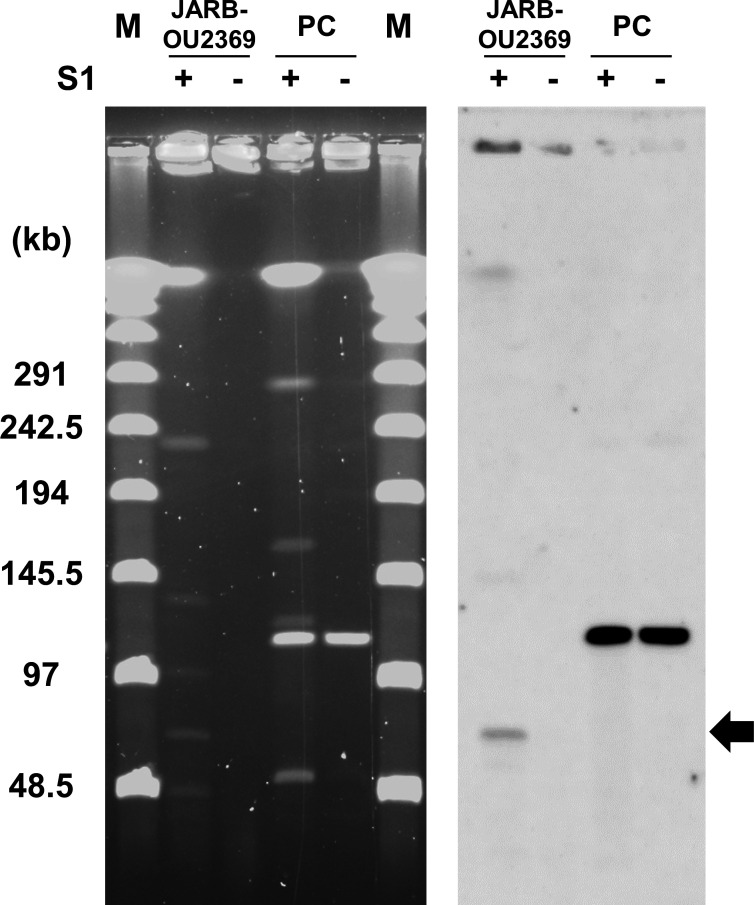
S1-PFGE for the *vanA*-positive plasmid in the Hiroshima isolate. S1-PFGE was performed using a Hiroshima *vanA*-positive isolate (JARB-OU2369) and a linear *vanA*-positive strain (S477) as the positive control (26). The image on the left shows staining with GelGreen (Biotium), and the image on the right shows a Southern blot using a *vanA* probe. The black arrow indicates *vanA*-positive plasmid fragments detected in JARB-OU2369.

The *vanA*-positive plasmids detected in the Hiroshima isolates were similar to the Indian-derived plasmid pA10290, which was reported to be a linear plasmid (19). However, the complete nucleotide sequence of *vanA*-positive pA10290-like plasmids of three Hiroshima strains was predicted to be circular. Therefore, we performed S1- pulse-field gel electrophoresis (PFGE) and Southern blotting to determine whether the plasmids were linear or circular (Fig. 6). If a plasmid is linear, the DNA fragment of the plasmid should be of the same size with and without S1 nuclease treatment. In the Hiroshima strain, a DNA fragment of *vanA*-positive plasmids was detected only when S1 nuclease treatment was applied and not without enzyme treatment, suggesting that the pA10290-like plasmid in the Hiroshima strain is circular (Fig. 6).

## DISCUSSION

Our genetic and phylogenetic analyses of clinical isolates from the VRE outbreak across two cities in Hiroshima between 2018 and 2021 showed that it was caused by a strain of *E. faecium* ST80 that was likely derived from A10290, a strain carrying the *vanA*-positive plasmid pA10290 first isolated in India (Fig. 1 to 3; Fig. S2; Table S1). Isolate ST 2441 in 2018, isolate ST78 in 2019 at the beginning of the outbreak, and isolate ST78 in 2021 appear to be sporadically detected strains because pA10290 was not detected in them (Fig. 2; Table 1; Table S1). *E. faecium* ST80 JARB-OU2378 was similar to A10290 and was first isolated in 2020; however, the carrier had no history of traveling abroad. In addition, the inferred transmission routes among hospitals were useful because the patient transfer histories corresponded to the reconstructed route; however, the routes of hospitals C, D, and F could not be inferred from other hospitals in Hiroshima (Fig. 4), suggesting that some transmissible strains may have been missed.

Outbreaks caused by ST80 have been reported worldwide (7–11, 15–19). In Denmark, multiclonal ST80s that carried the same plasmid harboring *vanA* have spread. In Ireland and India, ST80 is a predominant ST and multiclonal ST80s carry circular or linear plasmids containing *vanA* (12). In Japan, an outbreak with multiple clones of ST80 and ST992 occurred in Nara Prefecture in 2019 (27) but was not genomically analyzed; therefore, it could not be compared to the ST80 isolated in this study.

A10290 carries a *vanA*-positive plasmid and acquired vancomycin resistance. In this study, 100 of 103 VRE isolates carried a *vanA*-positive pA10290-like plasmid (Table 1). We determined the complete sequences of *vanA*-plasmids from 13 representative strains from each hospital. Twelve *vanA*-plasmids were similar to pA10290, and only one, pJARB-OU2599, was larger (318,750 bp, accession number LC799795). A comparison of *vanA*-plasmids suggested that the pA10290-like plasmid was fused with the pA10290_L-like plasmid in JARB-OU2599 (Fig. 5; Table S1). We also found an IS256 family transposase near the fused region, suggesting its involvement in recombination (Fig. 5). Moreover, combined with the existence of other recombined regions (Fig. 5), the sequence suggested that several recombination events might have occurred within the bacterial cells.

The pA10290 is reported to be a linear plasmid (19), and we expected the *vanA*-positive plasmid of Hiroshima to be linear as well. However, the results of the genome assembly and conjugation experiments raised two questions. First, the Flye and Unicycler assembler made the sequences circular, and second, no conjugation occurred in the broth during the mating experiments of *E. faecalis* or *E. faecium*. The linear plasmids pELF1 and pELF2 were detected in Japan, which have been reported to be highly transmissible not only on filters but also in broth (25, 28). In this study, the pA10290-like plasmid in the Hiroshima isolates was transferred via mating on the filter over ST and bacterial species (Table 2) but not in the broth, implying that it was not linear. The pA10290 plasmid might be linear; however, the original study on pA10290 did not provide additional validation other than its assembly analysis (19). Confirmation using S1-PFGE or other methods is necessary to characterize the pA10290 plasmid.

Upon examining the transfer frequency of the pA10290-like plasmid by conjugation using the Hiroshima isolate plasmid as the donor, the frequency was found to differ depending on the recipient species (Table 2), with high frequencies (1.1 × 10^−4^ to 1 × 10^−5^) reported for the same species of *E. faecium* regardless of ST (Table 2). Notably, *E. gallinarum* also showed a high frequency (1.1 × 10^−4^), while *E. faecalis* and *E. avium* had low frequencies (1.8 × 10^−6^ and 0.8 × 10^−7^, respectively; Table 2). Furthermore, it is unclear why *E. gallinarum* was more likely to accept the pA10290-like plasmid; the results may suggest that it has the potential to acquire foreign DNA. Moreover, *E. gallinarum*, being a commensal bacterium in healthy individuals, is considered a potential risk by serving as a reservoir for plasmids such as pA10290, thereby facilitating their propagation. The results of the conjugation transfer indicated that the pA10290-like plasmid could be transferred across STs and bacterial species, raising concerns regarding the widespread use of vancomycin-resistant genes.

Our study had several limitations. First, the collected VRE strains were only those that caused infections or were obtained through bacterial surveillance. When determining the transmission routes of these strains across hospitals, several strains with uncertain routes were observed (Fig. 4), suggesting that there may have been more strains that were not obtained during this period. Additionally, the measurement equipment used to determine vancomycin susceptibility in enterococci varied among medical institutions, which could have led to misidentification of some strains as non-VRE. To address this issue, it is necessary to implement proactive screening for VRE, such as screening for VRE upon hospital admission.

To the best of our knowledge, this is the first report to present a genetic and phylogenetic analysis of a large outbreak that has extended beyond the jurisdiction of health authorities in Hiroshima and was caused by a clonal strain of vancomycin-resistant *E. faecium* ST80, which was derived from the A10290 strain that was isolated in India. According to the inferred transmission routes across hospitals, the outbreak initially started from a general hospital with fewer than 500 beds, spread to multiple hospitals through a core general hospital, and eventually reached small- and medium-sized general or nursing care hospitals (Fig. 4). Patients are often transferred to chronic care hospitals or elderly care facilities after completing treatment in acute care hospitals. In this context, asymptomatic patients may be transferred as carriers of VRE, thereby spreading VRE to many hospitals. An outbreak like the current one occurred in Aomori in 2019 with ST1421 (23). Frequent occurrences of such outbreaks can result in high costs for countermeasures, potentially limiting the regional healthcare system (1). Therefore, thorough infection control measures at the central hospital serving as a hub and communication between hospitals in a collaborative relationship are crucial to bring the outbreak to an early resolution. Furthermore, the *vanA*-positive pA10290-like plasmid of the clonal strain spread to other *Enterococcus* species via conjugation. The spread of ST80, as well as the high frequency of conjugation transmission of the *vanA* plasmid to various *Enterococcus* species, suggests that careful attention should be paid to prevent the spread of VRE.

## MATERIALS AND METHODS

### Bacterial isolates

Between 2018 and 2021, 103 VRE (minimum inhibitory concentration of vancomycin, ≥16 mg/L; infection: 50 strains; colonization: 52 strains, environment: one strain) were isolated from patients or were provided through bacterial surveillance at 15 hospitals in Hiroshima and Hatsukaichi cities in Japan (Table S1; Fig. 1 and 2). Hiroshima city (population of 1,189,149 on December 31, 2021) is the capital of Hiroshima Prefecture, and Hatsukaichi city (population of 116,661 on December 1, 2021) is located to the west of Hiroshima city. The maximum distance between hospitals in these cities is approximately 20 km (Fig. 1). In January 2021, *vanA*-positive VRE were detected in multiple patients at Hospital G in Hiroshima city, which was considered an outbreak of VRE, and isolates from Hiroshima Prefecture from 2018 to 2021 were collected. These isolates were grown in Tryptic Soy Broth (TSB; BD, NJ, USA) at 37°C, and antibiotics were used for selection at the following concentrations: 20  µg/mL vancomycin and 10  µg/mL erythromycin.

### Whole-genome sequencing

Genome sequencing was performed as previously described, with some modifications (29, 30). Briefly, bacterial cells were grown in TSB for 16 h at 37°C and harvested at 13,000 *× g* for 2 min. The pellets were suspended in 0.2 mL of buffer (100 mM Tris-HCl [pH 7.5], 150 mM NaCl, and 10 mM EDTA) containing 5 mg/mL lysozyme (Fujifilm Wako Pure Chemical Corporation, Osaka, Japan) and 0.5 mg/mL RNase (Nippon Gene, Tokyo, Japan). After incubation at 37°C for 24 h, 0.5 mg/mL proteinase K (Nacalai Tesque, Kyoto, Japan) and 0.5% sodium dodecyl sulfate were added, followed by incubation at 55°C for 3 h. For short-read sequencing, genomic DNA was purified from the lysates using AMPure XP (Beckman Coulter, CA, USA), and short-read DNA libraries were prepared as previously described (31). Paired-end sequencing (2 × 150 bp) was performed using the Illumina HiSeq X Five platform (Macrogen Japan Corporation, Tokyo, Japan). For long-read sequencing, genomic DNA was purified using the Monarch HMW DNA Extraction Kit for Tissue (#T3060; New England BioLabs, Ipswich, MA, USA) following the manufacturer’s instructions. A long-read DNA library was prepared using the SQK-RBK004 rapid barcoding kit [Oxford Nanopore Technologies (ONT), Oxford, UK], and sequencing was performed on GridION (ONT) using MinKNOW v21.05.25 and a FLO-MIN106 flow cell (ONT).

### Analysis of whole-genome sequences

For *de novo* assembly, Illumina reads were trimmed using fastp v0.23.2 (32). Long reads were trimmed using Filtlong (https://github.com/rrwick/Filtlong), assembled with the trimmed long reads using Unicycler v0.5.0 (33) or Flye assembler v2.9.1 (34) and polished with trimmed Illumina reads using pilon v1.24 (35). Automated genome annotation was performed using the DFAST v1.2.18 prokaryotic genome annotation pipeline (36), and antimicrobial resistance genes were identified using Abricate v0.8 (https://github.com/tseemann/abricate) and the CARD database (37). The ST of the isolates was identified using MLST v2.11 [PubMLST database https://pubmlst.org/, https://github.com/tseemann/mlst (38)].

### Core genome MLST

The core genome MLST profile of each genome was generated using chewBACCA v2.8.5 (39) based on the 4,345 loci scheme. The scheme was generated using the CreateSchema module of chewBBACA with default settings using FASTA files of the Hiroshima isolates and sequence reads of *E. faecium* ST80 deposited in the NCBI (Table S2). The training file was downloaded from GitHub (https://github.com/B-UMMI/chewBBACA/blob/master/CHEWBBACA/prodigal_training_files/Enterococcus_faecium.trn). Allele calling from the core genome was performed using all genomes as previously described (40). A minimum spanning tree was constructed using PHYLOViZ Online (https://online.phyloviz.net/index).

### Phylogenetic analysis

Snippy v4.6.0 (https://github.com/tseemann/snippy) was used for phylogenetic analysis of organisms isolated from Hiroshima and India. SNPs were obtained between the isolates and the reference genome A10290 with default settings, and a core SNP alignment was generated using the snippy-core function. Gubbins v2.4.1 (41) was used to remove recombination from the resulting alignment, and snp-dists v0.8.2 (https://github.com/tseemann/snp-dists) was used to calculate pairwise SNP distances in the final alignment between all sequences. Phandango (42) was used to generate a maximum likelihood phylogenetic tree based on the alignment with metadata.

### Genome alignment to *vanA*-positive plasmid pA10290

To determine whether the isolates from Hiroshima harbored a *vanA*-positive plasmid related to pA10290 derived from India, Illumina reads from the isolates were aligned to the DNA sequence of pA10290 and pA10290_L using Bowtie2 with default settings (43). The output sequence alignment map file was converted into a binary alignment map file, sorted, and indexed using SAMtools v1.14 (44). Isolates with aligned reads covering over 90% of the reference sequence were defined as carrying the pA10290-like plasmid.

### Reconstruction of transmission within the outbreak

The direction of *E. faecium* ST80 transmission among hospitals in Hiroshima was inferred using the Bayesian approach BadTrIP, an open-source package for the Bayesian phylogenetic software BEAST2 (45, 46). BadTrIP requires epidemiological and genetic data. Genetic data comprised the nucleotide counts of SNPs at a specific position in each sample. Alignment data were generated from the core SNP genome output alignment file, analyzed with Snippy-Gubbins, and converted to count files using FastaToCounts.py of the PoMo script (https://www.biorxiv.org/content/10.1101/016360v2).

For epidemiological data, the date of each strain isolated from a patient was defined as the collection date, each hospital was defined as a host, and the host exposure duration was defined as the time between the first and last days of isolation of *E. faecium* ST80 from each hospital. Transmission during the outbreak was reconstructed based on a previous report (47).

### Conjugation assay

Conjugation assays were performed using filter mating, as previously described with some modifications (25). JARB-OU2369 (*E. faecium* ST80) was used as the donor strain, and JARB-HU0742 (*E. faecium* ST17), JARB-HU0749 (*E. faecium* ST547), JARB-HU0782 (*Enterococcus avium*), JARB-HU0796 (*Enterococcus faecalis* ST476), and JARB-HU0816 (*Enterococcus gallinarum*) were used as recipients. Donors and recipients were grown in TSB for 16 h at 37°C. The cultures were subsequently diluted 50-fold in fresh TSB and incubated for an additional 4 h at 37°C. A 50-µL aliquot of the donor or recipients was mixed, collected onto a 0.22-µm nitrocellulose filter (Advantec, Tokyo, Japan), and incubated in TSA for 16 h at 37°C. After conjugation, the filter was washed with 1 mL of TSB, and the suspension was inoculated on TSA-supplemented erythromycin and vancomycin. Conjugation frequency was calculated as the number of transconjugants per donor cell.

### S1-Nuclease PFGE and Southern blotting

We performed S1-PFGE as described by Fujiya *et al.* (26) with some modifications to determine the topological form of *vanA* plasmids. The bacterial cells in agarose gel plugs were lysed with 2 mg/mL lysozyme for 48 h at 37°C and treated with 1 mg/mL proteinase K for 16 h at 55°C. The plugs were washed thrice in TE buffer (10 mM Tris-HCl/1 mM EDTA, pH 8.0) and incubated in distilled water for 30 min at 20°C, followed by incubation with 50 U S1 nuclease (TaKaRa, Shiga, Japan) for 30 min at 23°C. Reactions were stopped by incubation with 0.5 M EDTA (pH 8.0) for 5 min at 20°C. The plugs were rinsed in TE-8 buffer and incubated for 1 h at 4°C in 0.5 × TBE buffer (50 mM Tris, 45 mM boric acid, and 0.5 mM EDTA). Total DNA in the plugs was digested with S1 nuclease, loaded onto a 1% agarose gel, and run on a CHEF Mapper XA pulsed-field electrophoresis system (Bio-Rad, Hercules, CA, USA) at 14°C in 0.5 × TBE buffer for 15 h under the following conditions: auto algorithm, 20–250 kb molecular weight, and 6.0 V/cm. After electrophoresis, the agarose gel was stained with GelGreen Nucleic Acid Gel Stain (1/10,000 dilution; Biotium) for 1 h at 20°C and visualized using a WSE-5200A Printgraph 2M system (ATTO, Tokyo, Japan). The DNA fragments were transferred to a nitrocellulose membrane using a Genie blotter (iDEA, MN, USA), hybridized with a digoxigenin-labeled probe (Roche, Basel, Switzerland) specific to *vanA*, detected using CDP-Star (Sigma-Aldrich, MO, USA) according to the manufacturer’s instructions, and visualized using an Amersham Imager 680 (Cytiva, MA, USA).

## Data Availability

The complete and annotated sequences were deposited in the public database of the DNA Data Bank of Japan under accession numbers AP027221-AP027238 and LC799788–LC799797. The short and long reads obtained from this study were deposited in DDBJ under the accession numbers BioProject PRJDB14951, BioSample SAMD00565567–SAMD00565669, and DRA accession DRR427953–DRR428058 and DRR530113–DRR530122.

## SUPPLEMENTAL MATERIAL

Supplemental material aac.01716-23-s0001.docx Tables S1 and S2; Fig. S1 to S3.

ASM does not own the copyrights to Supplemental Material that may be linked to, or accessed through, an article. The authors have granted ASM a non-exclusive, world-wide license to publish the Supplemental Material files. Please contact the corresponding author directly for reuse.
