# Supplementary material for: Genomic analysis of inter-hospital transmission of vancomycin-resistant Enterococcus faecium sequence type 80 isolated during an outbreak in Hiroshima, Japan
Source: Antimicrob Agents Chemother. 2024 Mar 20;68(5):e01716-23. doi: 10.1128/aac.01716-23 (PMC11064488; doi:10.1128/aac.01716-23)
Supplement: Supplemental material — Tables S1 and S2; Fig. S1 to S3. [file aac.01716-23-s0001.docx]

**Table S1. Genomic characteristics of the Hiroshima isolates**

**Table S2. List of *E. faecium* ST80 from this study that were deposited in NCBI**


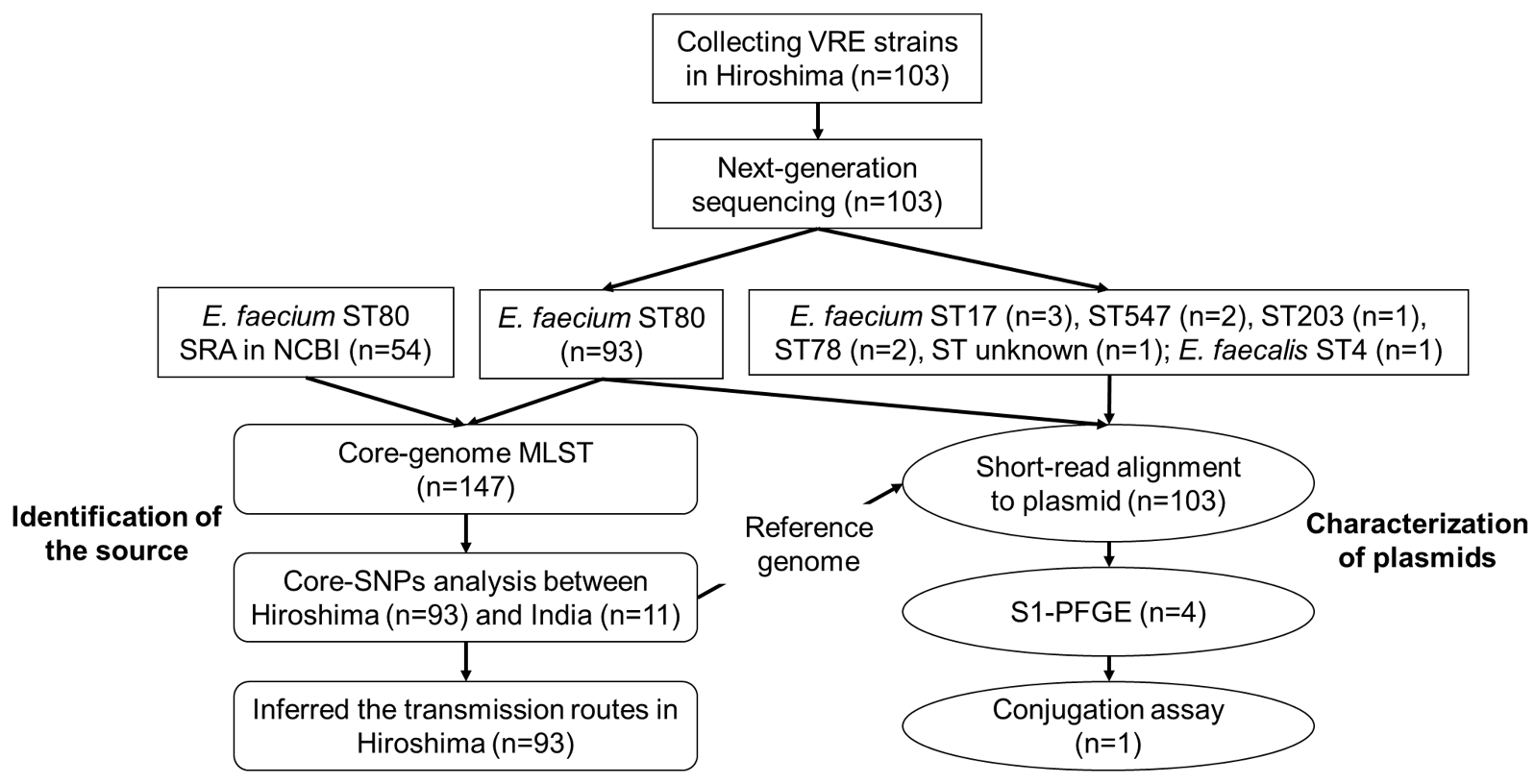


**Fig. S1 Flow chart of the analysis strategy for isolates in this outbreak.**

All 103 VRE isolates collected during the outbreak were subjected to whole-genome sequencing. Ninety-three of the 103 isolates were *E. faecium* ST80; therefore, genomic analysis was conducted to determine their source with the SRA of *E. faecium* ST80 deposited in the NCBI. Moreover, their plasmids were characterized to determine the vancomycin resistance mechanism of the 103 isolates.


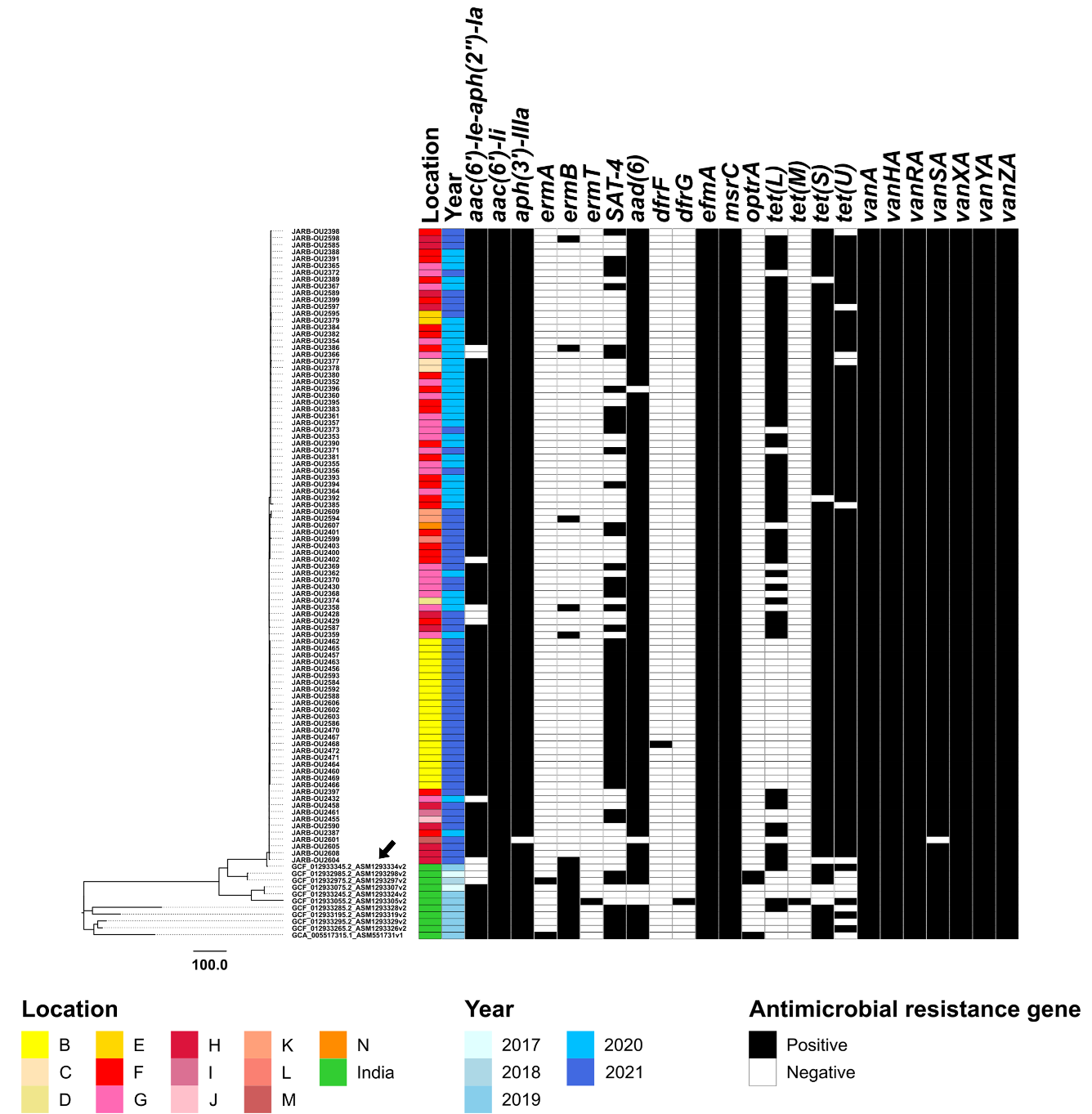


**Fig. S2 Single-nucleotide polymorphism analysis of *E. faecium* ST80 chromosomes isolated in Hiroshima and India**

A phylogenetic tree was generated by analyzing the core SNPs of the ST80 isolates from Hiroshima and the eight ST80 strains from India. The two-colored squares are next to the name of each strain, with the location of isolation indicated on the left and the year of isolation indicated on the right. The black arrow indicates the strain from India grouped within the Hiroshima clade.


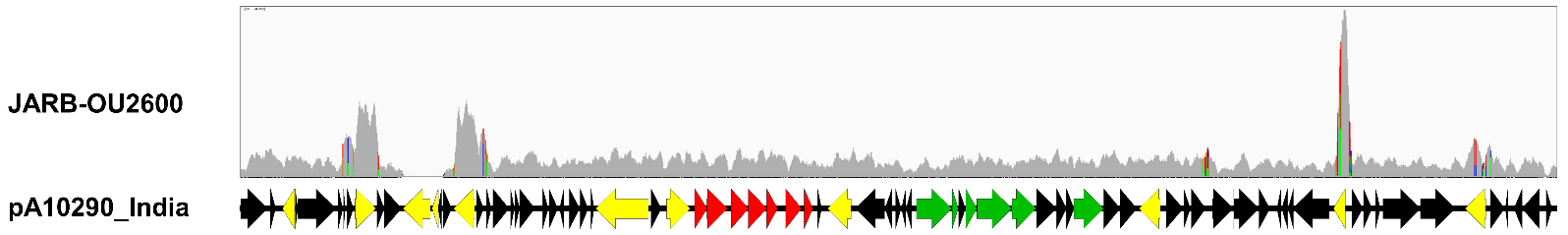


**Fig. S3 Mapping of the *vanA*-positive plasmid in *E. faecalis***

Only one strain of *E. faecalis* with *vanA*-positive plasmids was found among the Hiroshima isolates. The Illumina reads of the isolates were aligned using Bowtie2 and pA10290 as a reference and visualized using the Integrative Genomics Viewer. Gray indicates mapped reads, and red–blue–green lines indicate mutation sites.
